# The Relation of Hyperchlorhydria to the Motor Function of the Stomach1Read at the fortieth annual meeting of the Medical Association of Central New York, at Rochester, October 15, 1907

**Published:** 1908-01

**Authors:** Allen A. Jones

**Affiliations:** Adjunct Professor of Medicine Medical Department University of Buffalo


### The Relation of Hyperchlorhydria to the Motor Function of the Stomach[1]

By ALLEN A. JONES, M. D.

Adjunct Professor of Medicine Medical Department University of Buffalo

HYPERCHLORHYDRIA is best considered as a condition of high gastric acidity due to the presence of a large amount of free and combined hydrochloric acid, together with a sense of more or less discomfort, distress or pain in the stomach. Some individuals have a high gastric acidity, but are wholly uncon-

1. Read at the fortieth annual meeting of the Medical Association of Central New York, at Rochester, October 15, 1907

scious of it and make no complaint of disease of the stomach; while many others experience varying degrees of gastric discomfort, associated with what might be considered only a high normal acidity. I have at present under observation a young man whose total gastric acidity during digestion ranges from 70 to 110, with free hydrochloric acid .17% to .27%, but he has never complained of abnormal gastric sensations. As a clinical entity, however, we do not often find what may be termed a chemical unassociated with a sympathetic hyperchlorhydria. In other words, the stomach symptoms are urgent heralds of the existence of a high acidity.

There exists a gastric hyperesthesia as an imperative and important part of hyperchlorhydria. Irritative gastric symptoms are apt to dominate the clinical picture and these symptoms usually yield only to measures that either lower the acidity of the stomach contents or sedate the sensory innervation of the organ. It is commonly observed that a business or professional man, whose life is spent much indoors, develops the classic symptoms of hyperchlorhydria that disappear during the early days of his camping trip without a fall, but possibly a rise, in the acidity of his gastric contents. These ordinary clinical observations point to the existence of a strong sensory element in the complex which we term hyperchlorhydria. These constitute instances of the disease properly classed with the gastric neuroses. In these there need not exist an organic pyloric narrowing to account for the oft discovered tardy emptying of the stomach, because temporary pyloric spasm, due to high acidity of the contents of the stomach, constitutes a sufficient cause. The degree of pyloric spasm varies widely, and depends upon the percentage of gastric acidity, and upon the acuteness of local irritability of the pylorus, as well as upon general nervous erethism.

If hyperchlorhydric pylorospasm continues over a protracted period, pyloric thickening is apt to develop, and actual structural narrowing may thus be slowly brought about. The thickening may consist partly of muscular hypertrophy and partly of hyperemic or inflammatory hyperplasia of submucous and other tissues. There are clinical reasons for regarding hyperchlorhydria with its attendant pyloric states as highly predisposing to, if not really, excitive of, the development of gastric ulcer. It seems probable that the nutritive disturbance of the mucosa initiating ulcer may more easily manifest itself under these, than under quite normal, conditions. Be that as it may, it is an admitted clinical fact that ulcer is frequently found in these cases and if, in addition to the above mentioned tissue changes at the pylorus, there occurs contracting scar tissue, pyloric stenosis is well established.

The close relation between hyperchlorhydria and the motor function of the stomach is illustrated by this aspect of the subject. There are, however, a few points deserving of special discussion. In the first place, it is advisable, in order to clearly present the matter, to differentiate carefully between hyperchlorhydria, hypersecretion and continuous secretion (gastrosuccorrhea, Reichmann's disease). It has been definitely established by the observations of a very large number of workers, including myself, that there exists a secretory neurosis in which gastric juice of high acid value is secreted only during the time that stomach contains food and never otherwise. This is frequently found quite independently of delayed emptying of the stomach either because of pylorospasm or myoparesis. My observations are entirely confirmatory of Strauss's[1] contention, that the amount of contents withdrawn one hour after a test breakfast in these cases is usually but moderately greater than in the normal and that the increased quantity is due to a copious production of gastric juice and not to retention of the contents. Furthermore, in these cases of pure secretory disturbance no evidence of an abnormal condition of the pylorus is obtainable by chemic or microscopic examination of the gastric contents.

Regarding the advisability of using the terms hyperchlorhydria and hyperacidity interchangeably, I must dissent from the habit of some writers. Hyperacidity may depend upon hyperchlorhydria, upon retained gastric secretion, with or without fermentation acids in stagnating contents, or upon hypersecretion in a food-free stomach; as any degree of secretory acidity in an empty stomach may be strictly considered hyperacidity. For these reasons the term should not be used as a synonym for hyperchlorhydria. It is particularly important to determine as nearly as possible whether or not there is evidence of delay in the passage of the chyme through the pylorus in all cases of superacidity, and this can only be done by several examinations made at different hours after the test meals and especially by siphonage in the fasting stomach before breakfast.

If gastric juice is found in the fasting stomach the conclusion that it denotes pyloric narrowing is not warranted. Continuous secretion is doubtless, in some instances, an essential secretory neurosis, the etiology of which is but imperfectly understood. When, however, food remnants are present, even in very minute quantities, with gastric juice, there is warrant for strong suspicion of more or less stenosis and subsequent examinations should be made. There is so much likelihood of ulcer developing in these cases that the occult blood test should be conducted both as to feces and stomach contents; although a positive result of this test had best be considered as merely contributory information and not as settling the question of diagnosis.

In studying a large number of cases of persistent hyperchlorhydria, I have found several with pyloric narrowing that explained the protracted nature of the disease. The medical treatment of these cases is apt to prove disappointing, although much comfort may be given by rest, unirritating foods, alkalies with bismuth and local applications of ichthyol and rescorcin; yet some of these patients require operative treatment for the accomplishment of permanent cure. As a rule, pyloroplasty finds its most suitable place in these instances of partial stenosis, but there may be found sufficient thickening and deformity of the pyloric tissues to preclude this operation and force the surgeon to a gastro-enterostomy.

Some cases of hyperchlorhydria which I saw several years ago ago, have since returned with a history of almost continuous trouble, and I have found currants and other foods, given the previous night at dinner, present in the morning before breakfast, and have brought the patients to successful operation.

Casual mention is made of these, as they serve to illustrate and emphasise the manner in which a functional disorder of the stomach may either rapidly or gradually merge into a structural disease. A fact of some importance in this connection is observed in some cases presenting evidences of organic pyloric stricture, following a more or less prolonged period of irritative superacidity accompanied by the well-known symptoms of that complex. Whereas, in these cases, before the later organic changes develop the acidity is high, afterward low percentages prevail. Ewald[2] has recently emphasised the fact that hyperchlorhydria is by no means a constant and invariable accompaniment of gastric ulcer. Possibly late degenerative glandular changes supervene and partial secretory failure results.

In some patients presenting the associated phenomena, hyperchlorhydria and tardy emptying, it is quite probable an adequate explanation may exist in the propulsion of superacid chyme into the first portion of the duodenum. Pawlow's oft-quoted observation that pyloric relaxation depends upon neutralisation of that amount of acid chyme already in the duodenum, suggests the proposition that additional duodenal secretion and time are required to neutralise over acid contents, and thus there may be repeated delays in the more or less regular opening and closing function of the pylorus in hyperchlorhydria. It may be also that alkaline gastric sedatives, such as bicarbonate of soda, carbonate of magnesia and subcarbonate of bismuth hasten the propulsive function of the stomach as much through their effects in the first part of the duodenum as in the antrum pylori.

A question of interest and importance, though difficult of settlement, relates to the underlying causes of hyperchlorhydria. An attractive theory is that it may arise from an excess of a chemical stimulation of gastric secretion. The suggestive finding by Wertheimer and Popielski, that the secretion of pancreatic juice called forth by the presence of acid in the small intestine was wholly uninfluenced by division of both the vagus and splanchnics, by excision of the spinal cord, or by extirpation of the abdominal sympathetic, prompted Bayliss's and Starling's[3] experimental investigation that resulted in the finding of a substance named by them secretin, formed in and by the epithelia of the duodenal mucous membrane under the action of acids which, absorbed or injected into the blood, produces an active pancreatic secretion.

Edkins[4] experimentally determined that after the nervous connections of the stomach had been destroyed, the introduction into the jugular vein of the animal of a decoction made by boiling pyloric mucous membrane with acid, water or peptone, was followed in an hour by the secretion of an acid, proteolytic juice in the stomach. This substance is known as gastric hormone. Edkins concludes this to be the explanation of secondary gastric secretion or that occurring after so-called "appetite juice" is excited. Einhorn and I independently proved several years ago that direct electric stimulation of the mucous membrane of the stomach evokes the secretion of gastric juice in an empty stomach, therefore it would seem that secondary gastric secretion may not wholly depend upon chemical influence or control. It appears warrantable to look upon gastric secretion as being under the influence of several factors, viz: the brain through the thought, sight or smell of appetising food; the sympathetic by way of local stimulation of the mucous membrane of the stomach by foods, especially those that are thus active, and, lastly, the chemical substance named hormone, that operates independently of the nervous mechanism of the stomach.

Pathologically, hyperchlorhydria may possibly depend upon disturbance of one or more of these functions. It is suggestive that oils, which are usually not appetising, are said to lessen the secretion of hydrochloric acid; and they furthermore fail to form peptone, while they act as soothing agents locally to the mucous membrane of the stomach. Sailer and Farr[5] found that when oils were added to the food they had no inhibiting effect upon gastric digestion, but do not mention any observations upon its effect in the empty stomach. Their study, however, had particular reference to pepsin activity, and they made one observation of importance bearing upon the subject of my paper, and that was to the effect that excessively high gastric acidity, viz: 150, 145, and 136, exerted a decided inhibition toward peptic digestion. This may possibly account for a few of the cases of hyperchlorhydria in which proteids as well as carbohydrates show insufficient digestion and are present in the stomach too long after their ingestion.

Finally, let me emphasise the importance of having in mind, and searching for evidence of, a local area of hyperesthesia at or near the pylorus in all cases of hyperchlorhydria, as this area is apt to assume sooner or later the characteristics of a latent or active ulcer, and at some stage in the history of any given case symptoms of an organic lesion of the stomach may give place to that which heretofore had been considered a secretory and sensory neurosis.

Also it is well to remember the relation of other organic alterations of the pylorus to the symptom complex so frequently present in hyperchlorhydria. I have seen peripyloritis with peritoneal adhesions the cause of the disorder, and several cases of chronic cholelithiasis have exhibited both the symptoms and the gastric chemistry common to excess of hydrochloric acid. An important point in cases of ulcer and cholelithiasis, associated with high acidity of the stomach, consists in the frequently excessive character of the pain. It is not common to find severe pain with simple hyperchlorhydria, but there is more apt to be a sensation of burning or grawing in the epigastrium two or more hours after meals. With ulcer, food is apt to give rise to renewed pain, whereas with uncomplicated irritative overacidity food usually gives relief.

These are a few considerations that prompt careful investigation as to the organic state of the stomach and its adnexa in all cases of hyperchlorhydria.

1. Modern Clinical Medicine; Diseases of the Digestive System, page 106
2. Modern Clinical Medicine
3. The Physiology of Digestion, Starling, 1906
4. Journal of Physiology, Vol. XXXIV, Page 133, 1906
5. American Medical Sciences, Jan., 1907

436 Franklin Street.

